# MINA53 deficiency leads to glioblastoma cell apoptosis via inducing DNA replication stress and diminishing DNA damage response

**DOI:** 10.1038/s41419-018-1084-x

**Published:** 2018-10-17

**Authors:** Fan Xuan, Mengying Huang, Erhu Zhao, Hongjuan Cui

**Affiliations:** State Key Laboratory of Silkworm Genome Biology, Southwest University, 400716 Chongqing, China

## Abstract

MYC-induced nuclear antigen (MINA53) is a JmjC (jumonji C domain)-containing protein, which is highly expressed in many cancers including glioblastoma. We have revealed in our previous report that MINA53 is a poor prognostic indicator for glioblastoma patients, and knockdown of MINA53 could reduce glioblastoma malignancy. In this study, we found that MINA53 knockdown could decrease the DNA replication initiation in glioblastoma cells. Through further investigations, we revealed that MINA53 could regulate the expression of the CDC45-MCM-GINS (CMG) complex genes, which are vital for DNA replication initiation. Knockdown of MINA53 reduced the CMG genes expression and thus induced DNA replication stress and DNA damage. Furthermore, MINA53 knockdown diminished DNA damage response (DDR) by reducing the ATM/ATR-H2AX pathway activity and finally led glioblastoma cells to apoptosis and death. We further applied a genotoxic drug Doxorubicin and found that MINA53 deficiency sensitized glioblastoma cells to Doxorubicin. Our study reveals that MINA53 is involved in DNA replication initiation and DNA damage response, and provides support for MINA53 as a novel and potential therapeutic target for glioblastoma treatment.

## Introduction

MYC-induced nuclear antigen (MINA53^1^, also known as Mdig^2^, NO52^3^, RIOX2) is a JmjC (jumonji C domain)-containing protein which is highly expressed in many cancers, such as pancreatic cancer^4^, gastric adenocarcinoma^5^, lung cancer^6,7^, lymph cancer^8^, colon cancer^9^, esophageal squamous cell carcinoma^10^, neuroblastoma^11^, and cholangiocarcinoma^12^. In our previous report, we demonstrated that MINA53 is upregulated in glioblastoma and is a poor prognostic indicator; Knockdown of MINA53 significantly inhibits glioblastoma cell proliferation and tumorigenesis, which suggests that MINA53 could be a potential molecular target for glioblastoma therapy^13^.

DNA replication is a conserved cellular process which occurs exactly only once during cell-cycle progression for its initiation is tightly regulated^14^. The initiation of DNA replication can be divided into two steps: origin licensing and origin firing^15^. Origin licensing is the primary step which occurs in G1 phase. In this step, minichromosome maintenance protein (MCM) complex is recruited onto the origin DNA sequence to form DNA helicase^14,16–18^. In the process of origin firing, it is essential for DNA helicase to unwind double-stranded DNA at the replication fork. While the activation of DNA helicase depends on the stable association of MCM complex with cell division cycle 45 (CDC45) as well as Go-Ichi-Ni-San (GINS) complex to form the CDC45-MCM-GINS (CMG) complex^19^. The CMG complex is assembled by CDC45, MCM2~7 and GINS1~4, and each of these components is vital for the function of the complex^20^. The formation of CMG is indispensable for DNA replication initiation. Deficiency in one or more CMG genes would inhibit DNA replication initiation^21–23^.

Defective DNA replication initiation leads to under-replicated DNA and induces DNA replication stress and DNA damage^24,25^. Cells response to DNA damage by activating the DNA damage response (DDR) pathway, which can decide cell fate by driving the correctly DNA-repaired cell to survival or the cell failing to repair its DNA to death^26^. Ataxia telangiectasia-mutated (ATM) or ATM-Rad3-related (ATR) kinases are the key elements in DDR pathway, which can be recruited to the damage site and auto-phosphorylated. Then they phosphorylate and activate the essential downstream factor histone variant H2AX, producing γ-H2AX^27,28^. γ-H2AX is required for the assembly of DNA repair proteins at the DNA damage site^29^.

In this study, we established the functional relevance between MINA53 and DNA replication. We found that knockdown of MINA53 resulted in a decrease of CMG genes expression as well as an upregulation of H3K9me3 at CMG genes promoters. Less expression of CMG led to insufficient DNA replication initiation, and thus caused DNA damage. Furthermore, MINA53 knockdown inhibited the activation of ATM/ATR-H2AX pathway which has a key role in DNA damage response. The accumulating DNA damage and diminished DDR finally led glioblastoma cells to apoptosis and death. According to this, we further applied a broad-spectrum anti-tumor drug Doxorubicin and found that MINA53 knockdown sensitized glioblastoma cells to Doxorubicin, suggesting that MINA53 could be a potential therapy target for glioblastoma.

## Materials and methods

### Cell culture

Glioblastoma cell lines LN-229 and U-87 MG and human embryonic renal cell line 293FT were purchased from ATCC and cultured in Dulbecco’s modified Eagle’s medium (DMEM), supplemented with 10% fetal bovine serum (FBS) and 1% penicillin and streptomycin (P/S). 293FT growth medium contains extra 0.5 mg/mL G418, 4 mM l-glutamine, 0.1 mM non-essential amino acids (MEM), and 1 mM sodium pyruvate. The 293FT transfection medium do not consist P/S and G418. All cell lines were tested mycoplasma-negative and cultured in an incubator with 5% CO_2_ at 37 °C. The DMEM media, FBS, antibiotics, and supplements were purchased from Thermo Fisher (IL, USA).

### Transfection and infection

Lentiviral constructs expressing MINA53 short hairpin (sh) RNA (#1, TRCN0000130530; #2, TRCN0000291230) and the negative control non-mammalian plasmid (Control) (SHC002) were purchased from Sigma-Aldrich (MO, USA). The lentivirus was generated following the manufacturer’s instructions. Briefly, pLKO.1-shMINA53#1 or pLKO.1-shMINA53#2 vector, pLP1, pLP2 and pLP/VSVG plasmids were co-transfected into 293FT cells using lipofectermin^TM^2000. Viral supernatants were collected and clarified by filtration 48 h later. The lentivirus was used immediately or stored at −80 °C in small aliquots. Target cells were infected by lentivirus twice and then selected by puromycin (A1113802, Thermo Fisher, IL, USA) to gain the stable cell line.

### BrdU staining

Cells were seeded on coverslips, and incubated with 25 μM BrdU (B5002, Sigma-Aldrich, MO, USA) for 40 min, then washed with PBS and fixed in 4% paraformaldehyde (PFA) for 20 min. Subsequently, cells were treated with 1 M HCl and blocked with 10% goat serum for 1 h, and then incubated with BrdU antibody (ab6326, Abcam, MA, USA) overnight at 4 °C followed by Alexa FluorR 488 goat anti-rat IgG secondary antibody (H + L) (A-11006, Thermo Fisher, IL, USA) for 2 h at 25 °C. Nuclear was stained with 300 nM 4′, 6-diamidino-2-phenylindole (DAPI) (C1005, Beyotime, Shanghai, China) for 15 min at 25 °C. The percentage of BrdU-positive cells was calculated and 6 microscopic fields were counted (Nikon 80i, Nikon Corporation, Tokyo, Japan).

### DNA fiber assay

Overall, 2 × 10^3^ cells were seeded as a drop near the edge of one 100 mm plate and cultured overnight at 37 °C in 5% CO_2_ humidified incubator. The next day the cells were pulse-labeled with 25 μM iododeoxyuridine (IdU) (I7125, Sigma-Aldrich, MO, USA) and 250 μM thymidine analog chlorodeoxyuridine (CldU) (C6891, Sigma-Aldrich, MO, USA) for 15 min each. Then the medium was removed and cells were dried to sticky. When the cells were about to dry, they were overlaid with 20 μL lysis buffer (1 M Tris pH 7.4, 0.5 M EDTA, 10% SDS) for 10 min at 25 °C. Then the plate was tilted at 25° angle to let the buffer run to the bottom end of the plate so as to spread the DNA into fiber. The DNA fiber was air-dried and fixed in the solution with methanol: acetic acid as 3:1 for 10 min, then treated with freshly prepared 2.5 M HCl for 80 min at 25 °C. After that, the DNA fiber was blocked in 5% BSA in PBS for 30 min and then incubated with rat BrdU antibody (ab6326, Abcam, MA, USA) and mouse IdU antibody (ab181664, Abcam, MA, USA) overnight at 4 °C, followed by incubation with Alexa FluorR 594 goat anti-mouse IgG antibody (H + L) (A-11032, Thermo Fisher, IL, USA) and Alexa FluorR 488 goat anti-rat IgG antibody (H + L) (A-11006, Thermo Fisher, IL, USA) for 2 h at 25 °C. Images were acquired by Nikon (Nikon 80i, Nikon Corporation, Tokyo, Japan). The distance between active replication forks were measured using the ImageJ software.

### Quantitative reverse transcription-PCR (qRT-PCR)

The qRT-PCR experiments were performed as previously described^13^. The sequences of the primers used are listed in Supplementary Table S1.

### Western blot analysis

Cells were lysed and proteins were extracted. Then the proteins were separated by SDS-polyacrylamide gel electrophoresis, transferred onto polyvinylidene difluoride membranes, blocked with 5% defatted milk, and incubated with the primary antibodies overnight at 4 °C followed by the secondly antibodies at for 2 h 25 °C. The ECL SuperSignal West Femto kit (34096, Thermo Fisher, IL, USA) was used for visualization. The MINA53 antibody (397300, Thermo Fisher, IL, USA), γ-H2AX antibody (#9718), p-ATR antibody (#2853), p-ATM antibody (#5883), Cleaved-Caspase 3 antibody (#9664), and Cleaved-PARP antibody (#5625) were purchased from Cell Signaling Technologies (MA, USA). The H2AX antibody (ab124781), ATR antibody (ab2905), ATM antibody (ab32420) were purchased from Abcam (MA, USA). The GAPDH antibody (AF0006) was purchased from Beyotime (Shanghai, China).

### Chromatin immunoprecipitation (ChIP)-quantitative PCR (qPCR) (ChIP-qPCR)

ChIP assays were conducted using the EZ-ChIP^TM^ kit (17–371, Millipore, CA, USA) following the manufacturer’s instructions. Briefly, after sheared by sonication, the 200–1000 bps DNA fragments were immunoprecipitaed by MINA53 antibody (397300, Thermo Fisher, IL, USA) or H3K9me3 antibody (ab113754, Abcam, MA, USA), and then reverse cross-linked and purified. qPCR was conducted subsequently to determine the enrichment flod. The primer sequences used are listed in Supplementary Table S2.

### Comet assay

Cells were collected, embedded in a thin agarose gel on a microscope slide at a density of 1 × 10^5^ cells/mL and incubated with cell lysis buffer (2.5 M NaCl, 100 mM Na_2_EDTA, 10 mM Tris, 1% Triton and 10% DMSO) at for 2 h 4 °C, then pre-treated with electrophoresis buffer (0.3 M NaOH, 1 mM Na_2_EDTA) at for 15 min 25 °C to unwind the DNA. Electrophoresis was performed at 25 V, 300 mA for 20 min. Subsequently the DNA was incubated with 0.4 M Tris-HCl (pH = 7.5) at for 15 min 25 °C, and stained in ethidium bromide (EB) buffer for 20 min. Images were acquired by Nikon (Nikon 80i, Nikon Corporation, Tokyo, Japan).

### Immunofluorescence

Cells were cultured on coverslips and fixed in 4% paraformaldehyde (PFA) for 20 min. Subsequently, the cells were blocked with 10% goat serum with 0.1% Triton X-100 for 1 h at 25 °C, and then incubated with γ-H2AX antibody (#9718, Cell Signaling Technologies, MA, USA) overnight at 4 °C followed by Alexa FluorR 488 goat anti-rabbit IgG secondary antibody (H + L) (A-11034, Thermo Fisher, IL, USA) for 2 h at 25 °C. Nuclear was stained with 300 nM DAPI (C1005, Beyotime, Shanghai, China) for 15 min at 25 °C. The percentage of γ-H2AX-positive cells was calculated and 6 microscopic fields were counted (Nikon 80i, Nikon Corporation, Tokyo, Japan).

### Trypan blue staining

Trypan blue working solution (C0011, Beyotime, Shanghai, China) was added to the cell suspension (2 × 10^6^ cells/mL) as a proportion of 1:1. After mixed uniformly, the cells stained or not were counted using a hemocytometer.

### Flow cytometric apoptosis assays

The experiments were performed with an Annexin-V-FITC apoptosis detection kit (C1063, Beyotime, Shanghai, China) following the manufacturer’s instructions. Briefly, cells were collected and washed with PBS, gently resuspended in the 1 × binding buffer and incubated with Annexin-V-FITC/PI. An Accuri C6 Flow Cytometer (BD, NJ, USA) was applied to analyze the data following the manufacturer’s instructions.

### Statistical analysis

Experiments were carried out in 3 or more technical and biological replicates. Statistical analysis was performed as previously described^13^. Statistical parameters are specified in the figure legends. Data are presented as mean ± s.e.m., and *P* < 0.05 was considered statistically significant.

## Results

### Downregulation of MINA53 decreases DNA replication initiation in glioblastoma cells

**Fig. 1:**
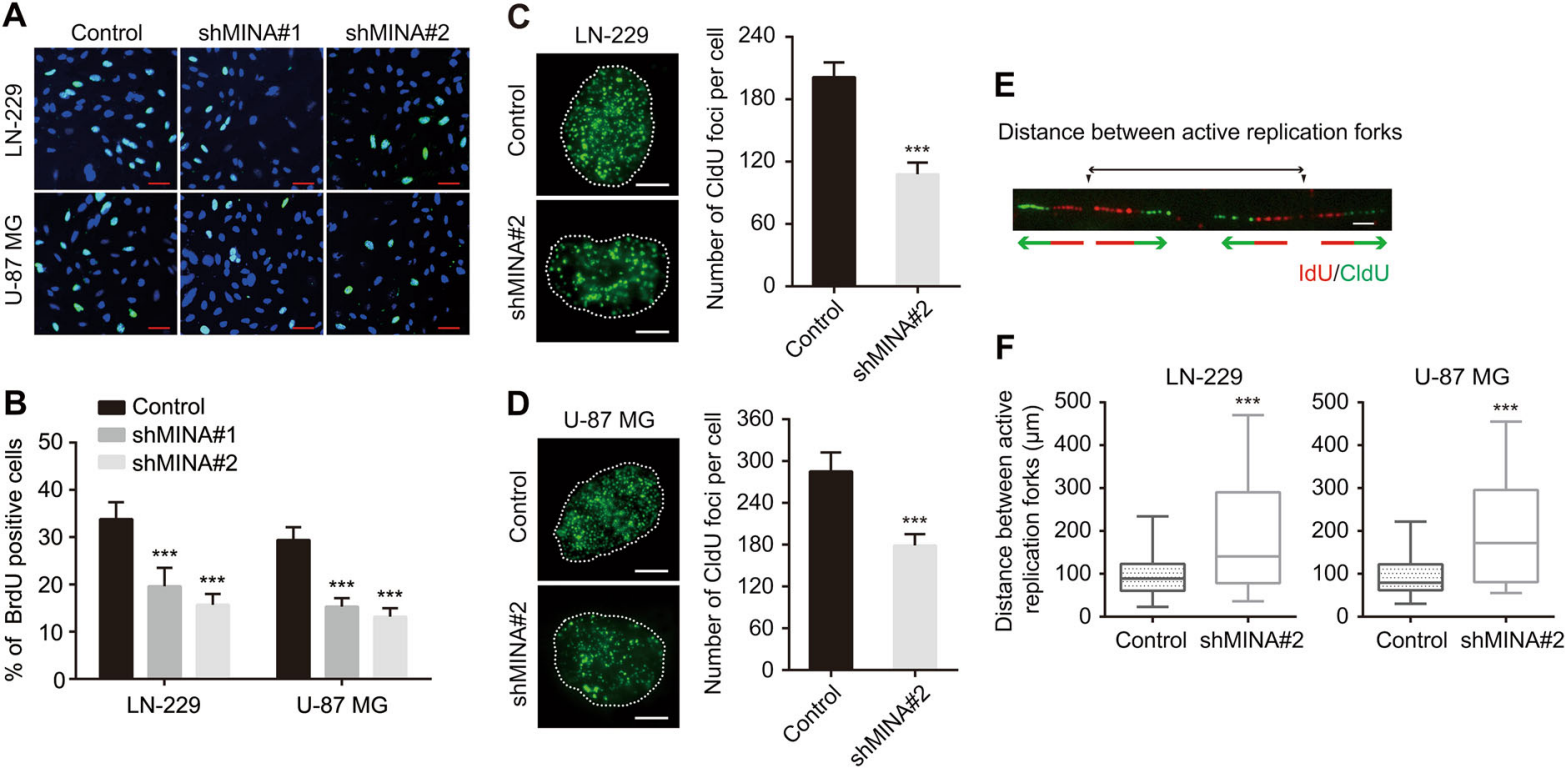
Downregulation of MINA53 decreases DNA replication initiation in glioblastoma cells. **a** Representative fluorescent micrographs of BrdU assays and **b** quantification of BrdU-positive cells of LN-229 and U-87 MG cells expressing Control, shMINA53#1 or shMINA53#2, Scale bar = 10 μm, *n* = 6. **c**, **d** Representative fluorescent micrographs of CldU assays and quantification of the number of CldU foci per indicated cell. Scale bar = 2 μm. *n* = 20. **e** Diagram of the distance between active replication forks. **f** Quantification of the distance between active replication forks. *n* = 20. All data were analyzed using two-tailed Student’s *t*-tests. Mean ± s.e.m., ***P < 0.001

In our previous report, we found that knockdown of MINA53 resulted in glioblastoma cell cycle arrest in G1 phase, suggesting a potential function of MINA53 in DNA replication^13^. To explore further of this, we established four stable MINA53-knockdown glioblastoma cell lines (LN-229 shMINA53#1, LN-229 shMINA53#2, U-87 MG shMINA53#1, and U-87 MG shMINA53#2) and performed a BrdU incorporation assay. We found that the percentage of BrdU-positive cells were significantly decreased in the MINA53-knockdown groups in comparison with the control groups both in LN-229 and U-87 MG cell lines (Fig. 1a, b). Next, to investigate whether MINA53 knockdown caused a direct hindrance in the execution of the DNA replication program, we quantified the number of the replication foci which correlates with the number of active replication clusters^30^. Cells were incubated with the CldU for 15 min and fixed, and then CldU was detected by immunostaining. We quantified the number of CldU foci per cell and found that the MINA53-knockdown groups displayed lower number of replication foci (CldU foci) than the control groups in both LN-229 and U-87 MG cell lines (Fig. 1c, d). Besides, we also performed a DNA fiber analysis to measure the distance between adjacent active replication forks within the replication clusters. Cells were sequentially incubated with IdU and CldU for 15 min each and DNA fibers were spread out. IdU and CldU were detected by immunostaining using specific antibodies. The distance between adjacent active replication forks was calculated (Fig. 1e). And we found that the MINA53-knockdown cells exhibited an average increase on the distance between adjacent active replication forks (Fig. 1f). These results suggested that knockdown of MINA53 decreases DNA replication initiation.

### MINA53 deficiency inhibits the CMG complex genes expression

**Fig. 2:**
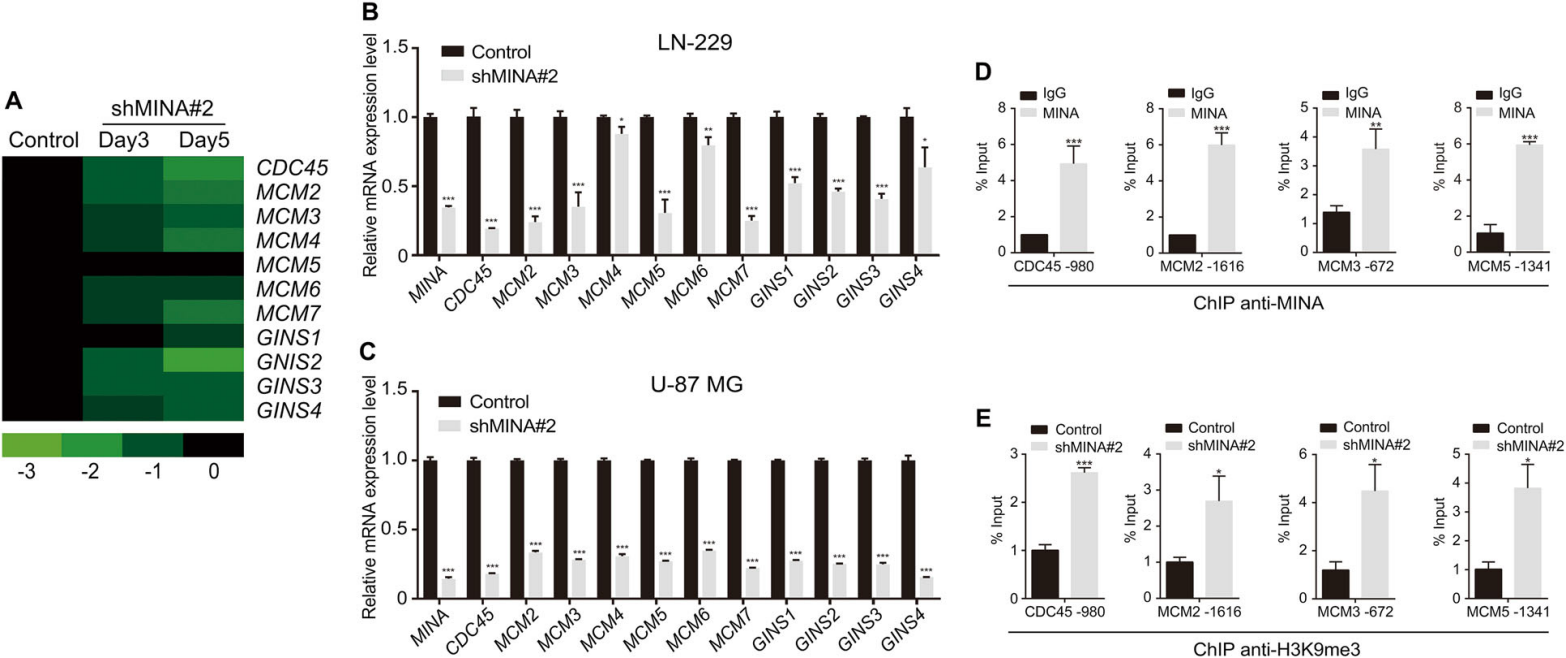
MINA53 regulates the CMG complex genes expression. **a** Expression alteration of CMG genes in LN-229 cells after MINA53 knockdown for 3 or 5 days. **b** mRNA expression of the indicated genes in LN-229 and **c** U-87 MG cells with or without MINA53 knockdown. **d** ChIP-qPCR analysis of MINA53 enrichment at the promoter regions of *CDC45*, *MCM2*, *MCM3*, or *MCM5* in LN-229 cells. **e** ChIP-qPCR analysis of H3K9me3 enrichment at the promoter regions of *CDC45*, *MCM2*, *MCM3*, or *MCM5* in LN-229 cells with or without MINA53 knockdown. All data were analyzed using two-tailed Student’s *t*-tests. Mean ± s.e.m., *n* = 3, *P < 0.05, **P < 0.01, ***P < 0.001

To further investigate how MINA53 expression is linked to DNA replication initiation, we examined our microarray data^13^ and found that the expression of a set of genes (*CDC45*, *MCM2~* *7*, *GINS1~* *4*) encoding the proteins of the CMG complex were significantly down-regulated after knockdown of MINA53 (Fig. 2a). qRT-PCR analysis further confirmed the results (Fig. 2b, c). We also examined the protein levels of some CMG complex proteins (CDC45L, MCM2, MCM3 and MCM5) using western blot analysis and found that they were consistently down-regulated (Supplementary Fig.S1). The CMG complex is a conserved component of the DNA replication system and is indispensable for the DNA replication initiation^21,31^. Deficiency in one or more CMG complex genes would inhibits DNA replication^21–23^. Our data showed that MINA53 deficiency inhibited the expression of the CMG complex genes, suggesting that MINA53 may correlate with DNA replication through altering the expression of the CMG complex.

### MINA53 knockdown increases H3K9me3 level at CDC45, MCM2, MCM3, and MCM5 promoters

Changes in the expression of MINA53 is usually accompanied with H3K9me3 alteration^13,32^. According to this, we performed a chromatin immunoprecipitation (ChIP) assay to determine whether the downregulation of CMG genes was associated with H3K9me3 alteration. As shown in Fig. 2d, we found a high enrichment of MINA53 in the promoter region of *CDC45*, *MCM2*, *MCM3*, and *MCM5*. In addition, after knockdown of MINA53, a significant increase of H3K9me3 level was detected in their promoter region where MINA53 was enriched (Fig. 2e). Tri-methylation on H3K9 tends to suppress gene transcription^33^. Our data show that knockdown of MINA53 increased the level of H3K9me3 on *CDC45*, *MCM2*, *MCM3*, and *MCM5* promoters and thus decreased their expression in vitro.

### MINA53 deficiency induces DNA damage and reduces the activity of the ATM/ATR-H2AX pathway

**Fig. 3:**
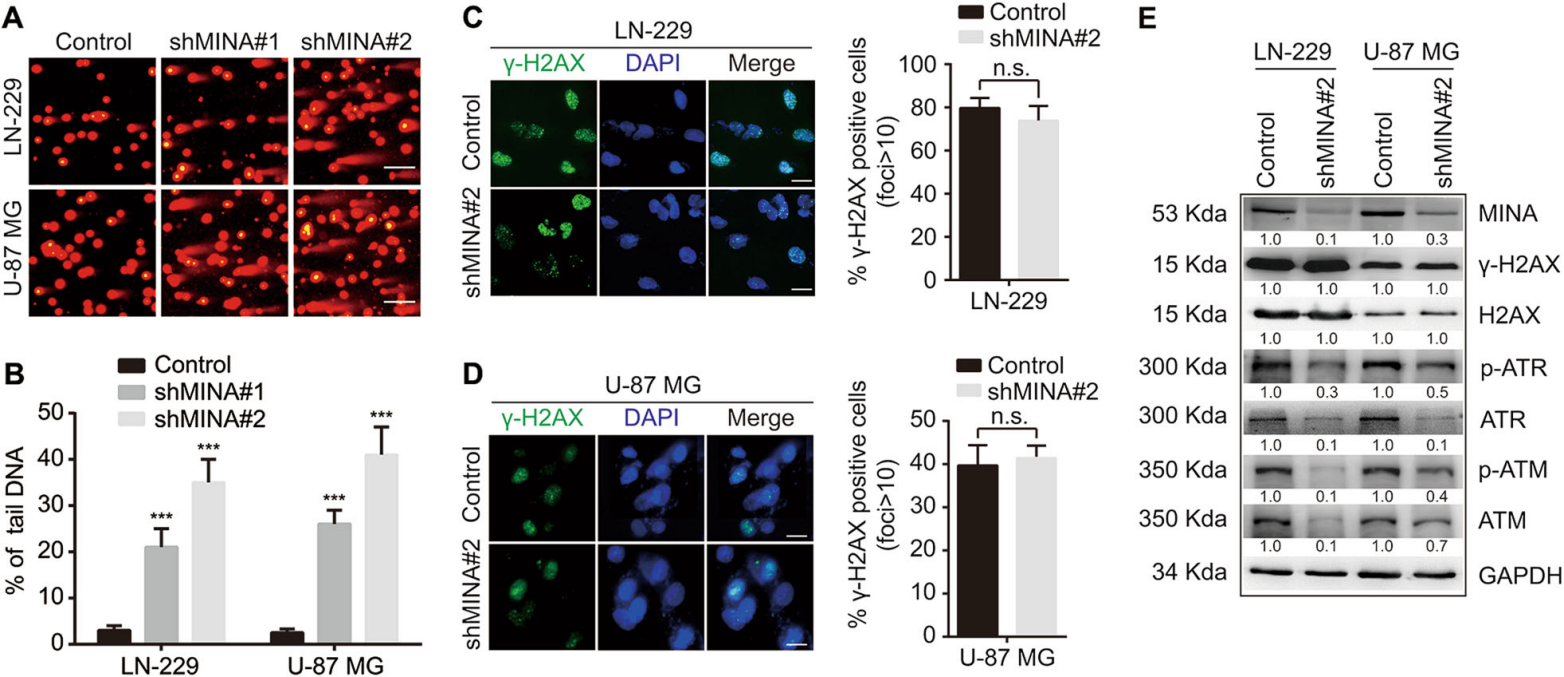
MINA53 deficiency induces DNA damage and reduces the activity of the ATM/ATR-H2AX pathway. **a** Representative fluorescent micrographs of comet assays and **b** quantification of the percentage of tail DNA of the indicated cells. Scale bar = 15 μm. **c**, **d** Immunofluorescence staining of γ-H2AX and quantification of γ-H2AX-positive cells of the indicated cells. Scale bar = 5 μm. **e** Western blot analysis of the indicated proteins in LN-229 and U-87 MG cells with or without MINA53 knockdown. All data were analyzed using two-tailed Student’s *t*-tests. Mean ± s.e.m., *n* = 3, ***P < 0.001

Cells that have deficiency in DNA replication initiation are easy to accumulate DNA lesion^14,22,25,34,35^. We further investigated whether the DNA replication initiation decrease caused by MINA53 knockdown would lead to DNA damage. We performed a comet assay, a technique to measure DNA damage in an individual cell using single-cell gel electrophoresis. We collected the cells 5 days after MINA53 knockdown, embedded them in a thin agarose gel on a microscope slide and then carried out the electrophoresis analysis. We observed a great amount of tail DNA in the MINA53-knockdown groups but bare in the control groups (Fig. 3a, b), indicating that knockdown of MINA53-induced DNA damage in glioblastoma cells. The histone H2AX will be phosphorylated as a key signal for DNA damage response^36^, which is required for the assembly of DNA repair proteins at the sites containing damaged chromatin^29^. Hence, we further examined the H2AX phosphorylation status in glioblastoma cells after MINA53 knockdown using immunofluorescence. Interestingly, we found that the percentage of phospho-Serine 139 H2AX (γ-H2AX)-positive cells did not change much in the MINA53-knockdown groups compared to the control groups (Fig. 3c, d). Western blot analysis also demonstrated that γ-H2AX did not increase after knockdown of MINA53 (Fig. 3e). H2AX can be phosphorylated by ataxia telangiectasia-mutated (ATM) kinase or ATM-Rad3-related (ATR) kinase after they are activated by autophosphorylation in the DDR pathway^27,37,38^. Therefore, we detected the status of ATM and ATR kinases by western blot to investigate why the γ-H2AX did not increase when cells were suffering from DNA damage induced by knockdown of MINA53. We found that both ATM and ATR as well as their active form p-ATM and p-ATR were reduced significantly after MINA53 knockdown (Fig. 3e). These data demonstrated that knockdown of MINA53 reduced the expression and activity of ATM and ATR kinases and thus inhibited the H2AX to be phosphorylated, suggesting that MINA53 knockdown inhibits the ATM/ATR-H2AX pathway and thus diminishes DNA damage response in glioblastoma cells.

### Knockdown of MINA53 induces glioblastoma cell apoptosis

**Fig. 4:**
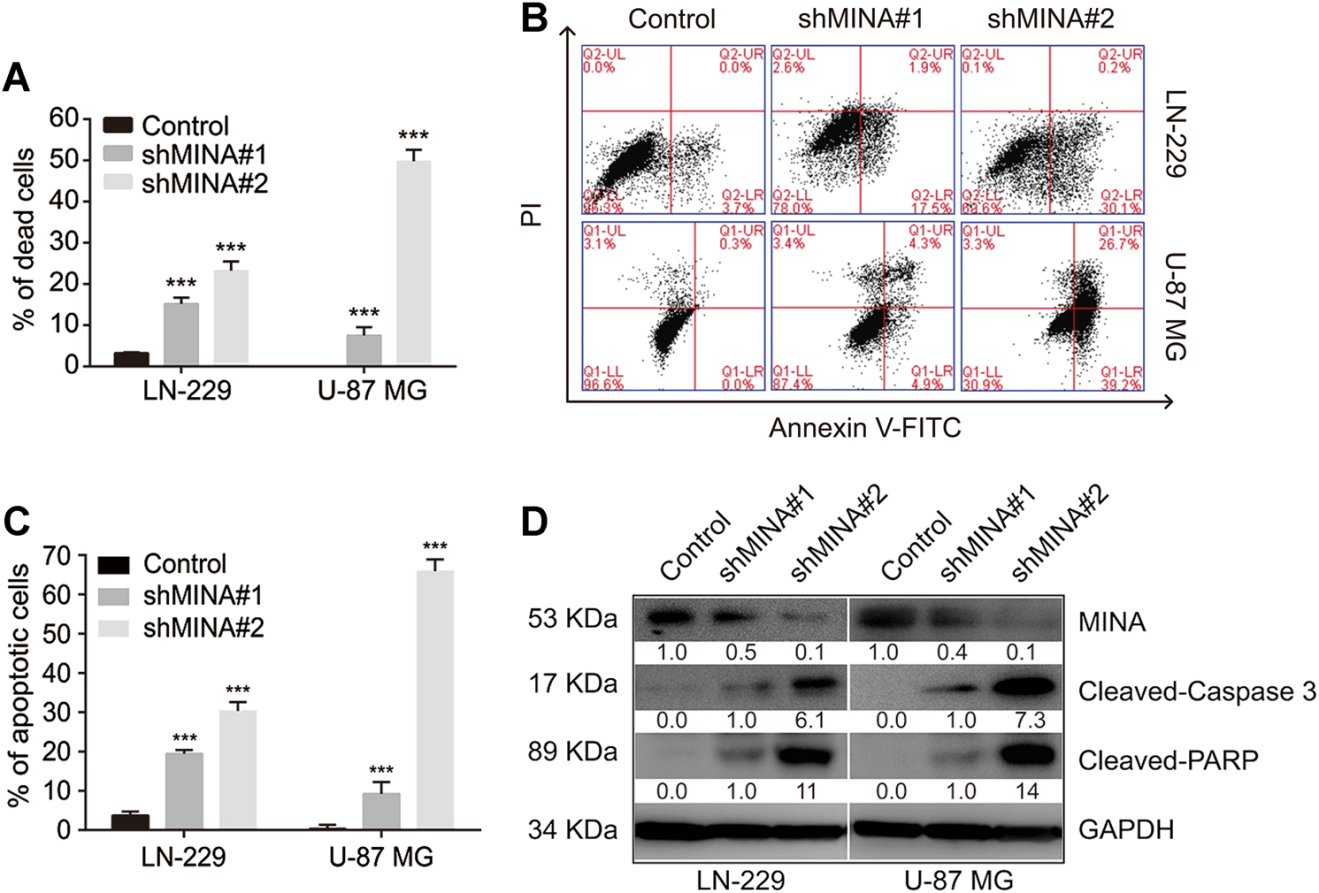
Knockdown of MINA53 induces glioblastoma cell apoptosis. **a** Quantification of the percentage of dead cells of the indicated cells. **b** Flow cytometry assays analyzing cell apoptosis and **c** quantification of the percentage of apoptosis cells of LN-229 and U-87 MG cells with or without MINA53 knockdown. **d** Western blot analysis of the indicated proteins in LN-229 and U-87 MG cells with or without MINA53 knockdown. All data were analyzed using two-tailed Student’s *t*-tests. Mean ± s.e.m., *n* = 3, ***P < 0.001, n.s. no significance

Suffering from DNA damage and diminished DDR is a great challenge to cell survival. We then explored whether knockdown of MINA53 would lead glioblastoma cell to die. We tracked the cells condition after knocking down MINA53, and found that 5–6 days later cells with MINA53 knockdown floated gradually, whereas the control cells did not. Subsequently we collected the cells and analyzed them using trypan blue staining and flow cytometry assay, respectively. Dead cells were stained with trypan blue and counted as well as the living cells. As shown in Fig. 4a, the MINA53-knockdown groups showed a greater proportion of dead cells than the control groups. In addition, we stained the cells with annexin-V-FITC and PI and then analyzed them with flow cytometry. The results showed that the number of apoptosis cells in the MINA53-knockdown groups increased significantly compared with the control groups (Fig. 4b, c). Moreover, we did a western blot analysis and found that Caspase 3 (a critical executioner of apoptosis) was activated (cleaved) and PARP (one of the main cleavage targets of Caspase 3) was cleaved after MINA53 was knocked down (Fig. 4d). These together with the above data suggested that knockdown of MINA53 in glioblastoma cells induces cell death through DNA damage-associated cell apoptosis.

### MINA53 deficiency sensitizes glioblastoma cells to Doxorubicin

**Fig. 5:**
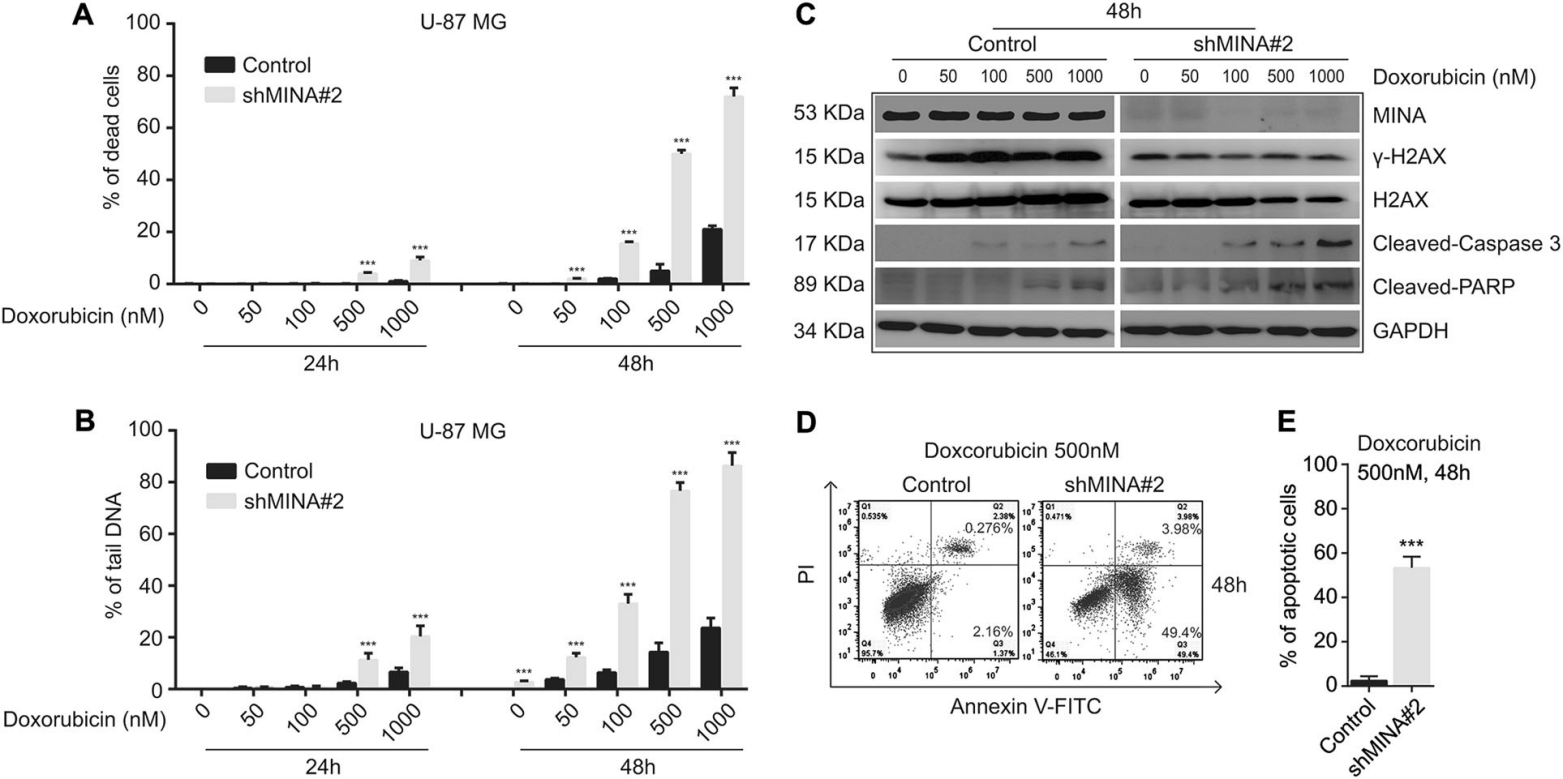
MINA53 deficiency sensitizes glioblastoma cells to Doxorubicin. U-87 MG cells with or without MINA53 knockdown were stimulated with Doxorubicin at indicated concentrations for 24 or 48 h. **a** Quantification of the percentage of dead cells and **b** the percentage of tail DNA in the indicated cells. **c** Western blot analysis of the indicated proteins after U-87 MG cells with or without MINA53 knockdown were stimulated with Doxorubicin at the indicated concentrations for 48 h. **d** Flow cytometry assays analyzing cell apoptosis and **e** quantification of the percentage of apoptosis cells of U-87 MG cells with or without MINA53 knockdown after stimulated with 500 nM Doxorubicin for 48 h. All data were analyzed using two-tailed Student’s *t*-tests. Mean ± s.e.m., *n* = 3, ***P < 0.001

DDR insufficiency makes cells sensitive to DNA damage. To further illuminate the relationship between MINA53 and glioblastoma cell DNA damage and cell death, we applied a DNA damage-inducing agent Doxorubicin (an efficient DSB inducing chemotherapy medication widely used in cancer) to study whether MINA53 deficiency would sensitize glioblastoma cell to it. In consistent with our previous report, 2 days after we knocked down MINA53 in glioblastoma cells the expression of MINA53 was efficiently down-regulated^13^. Considering that cell apoptosis was induced 5 days after MINA53 was knocked down, to avoid the following experiments being affected by the knockdown itself, we did the drug study 2–4 days after knockdown of MINA53. Cells were treated with Doxorubicin in a dose-dependent and time-dependent manner, and then the percentage of dead cells was counted by trypan blue staining. As shown in Fig. 5a, after treated with Doxorubicin, the MINA53-knockdown groups exhibited a much greater proportion of dead cells than the control groups, indicating that knockdown of MINA53 sensitized glioblastoma cells to Doxorubicin. Moreover, we examined the DNA damage status in the cells treated with Doxorubicin using comet assay, and observed more tail DNA in the MINA53-knockdown groups than the control groups (Fig. 5b), indicating that Doxorubicin induced more DNA damage in the MINA53-knockdown groups. We further checked the H2AX status and found that in each concentration Doxorubicin failed to increase the γ-H2AX level in the MINA53-knockdown cells, and the MINA53-knockdown cells exhibited a much higher level of Cleaved-Caspase 3 and Cleaved-PARP in comparison with the corresponding control cells (Fig. 5c). Furthermore, we conducted a flow cytometry apoptosis analysis and found that Doxorubicin induced more apoptotic cells in the MINA53-knockdown groups than their corresponding control groups (Fig. 5d, e). These results were consistent with our results above (Figs. 3e and 4d) and further demonstrated that knockdown of MINA53 sensitized glioblastoma cells to Doxorubicin; after treated with Doxorubicin, glioblastoma cells with MINA53 knocked down suffered severer DNA damage and diminished DDR and ultimately died through cell apoptosis.

## Discussion

DNA helicase is activated when the CMG complex is formed, which unwinds double-stranded DNA at the replication fork and is essential for the initiation of DNA replication^39–41^. Deficiency in one or more CMG genes would inhibit DNA replication initiation^21–23^, and insufficiency of DNA replication would cause DNA replication stress and DNA damage^24,25^. MINA53 is reported to show physical interaction with several DNA replication proteins (MCM7, MCM5, ORC5)^42^, which indicates that MINA53 may have a role in DNA replication. In this study, we provide evidence for the first time that MINA53 could regulate the expression of the CMG genes. Knockdown of MINA53 reduced the expression of *CDC45, MCM2~7* and *GINS1~4* genes (Fig. 2). And the reduction of the CMG genes resulted in the defective DNA replication initiation (Fig. 1) which contributed to the generation of DNA damage (Fig. 3a, b). Moreover, we found that MINA53 could bind to the promoter regions of several CMG genes *CDC45*, *MCM2*, *MCM3,* and *MCM5*. Knockdown of MINA53 would increase the H3K9me3 methylation level on these gene promoters. However, no effective enrichment of MINA53 was found on the promoters of other CMG genes (data not shown), indicating that MINA53 might affect the CMG genes expression through a more complicated way which needs to be further investigated. Overexpression of the CMG proteins have been observed in several malignancies^43–45^. Many studies demonstrate that downregulation of the CMG genes can have anti-cancer effect^22,25,34,46,47^. MINA53 deficiency would reduce the expression of the CMG genes, which gives inspiration that MINA53 could work as a molecular target for anti-cancer therapy.

Cells response to DNA damage by activating the DDR pathway, which can promote the correctly DNA-repaired cell to survive or the cell failing to repair its DNA to die^26^. In recent years, it has been well demonstrated that deficiency in DDR regulating genes is involved in tumorigenesis and cancer development. Defect of one or more DDR pathways during malignancy genesis trends to trigger DDR dependencies. Targeted therapies based on inhibiting the DDR in cancers offer a new strategy to fight against drug resistance^48^.

Wang et al provided evidence for the contribution of MINA53 to DDR. They showed an enhanced ATM phosphorylation by overexpressing MINA53 and a reduced ATM phosphorylation by down-regulating MINA53 in lung cancer cells treated with phleomycin^42^. In our study, we found that knockdown of MINA53 decreased not only the expression of ATM and ATR but also their phosphorylation pattern in glioblastoma cells. p-ATM and p-ATR are the active forms of ATM and ATR, which can phosphorylate H2AX to mediate the DDR. These could be a reason why we did not observe γ-H2AX upregulation after treated with Doxorubicin in MINA53 knockdown cells (Fig. 3–e).

Doxorubicin is widely used in cancer treatment as a chemotherapy genotoxic drug^49^. However, Doxorubicin is not commonly used in clinical glioma chemotherapy regimens due to it is less capable to pass through the blood–brain barrier. Nevertheless, development in interstitial chemotherapy makes it possible to deliver drugs directly at the site of brain tumor^50^, which provides more therapeutic options for patients who are suffering from brain tumor. Our study shows that knockdown of MINA53 sensitized glioblastoma cells to the genotoxic drug Doxorubicin, which gives more support for gene and drug combination therapeutics and may help to further improve the efficacy of glioblastoma interstitial chemotherapy.

c-Myc is a proto-oncogene that is usually implicated in glioblastoma. c-Myc can directly promote the proliferation of cancer cells, regulate the histone methylation, DDR, and DNA replication^51–53^. MINA53 is discovered as a Myc-induced nuclear antigen, and is directly regulated by c-Myc^1^. Researches reveal that MINA53 is over-expressed in many types of cancers^4,6,8,9,11–13,54,55^, and repressing MINA53 expression can efficiently suppress cancer cell proliferation. Besides, there are studies showing that MINA53 is involved in histone methylation^2,6,13,32^. Our results together with those previous studies give us a hint that MINA53 may be one of the important downstream genes of c-Myc, which has a significant role in regulation of cell proliferation, histone modification, DDR, and DNA replication.

Our data show that knockdown of MINA53 increased the level of H3K9me3 on several CMG gene promoters and thus decreased their expression in vitro. To further evaluate the relationship of MINA53 and CMG genes in vivo, we analyzed their expression level in two TCGA clinical databases: Glioblastoma (Cell 2013)^56^ and Brain low grade glioma (http://www.cbioportal.org/index.do). We compared the mRNA level of MINA53 in the two databases and found that MINA53 tend to have a higher mRNA expression in GBM than in low grade glioma. CDC45, MCM2, MCM3, MCM5 and ATR also have the same tendency, except for ATM (*P* = 0.34) (supplemental data 2). We also performed a linear regression analysis to see the relevancy of MINA53 expression with CDC45, MCM2, MCM3, MCM5, ATM, and ATR expression. We found a positive correlation between the mRNA level of MCM2 (*R*^2^ = 0.027, *P* = 0.039), MCM3 (*R*^2^ = 0.04, *P* = 0.01), ATM (*R*^2^ = 0.19, *P* < 0.0001), ATR (*R*^2^ = 0.26, *P* < 0.0001), and the mRNA level of MINA53, respectively. The mRNA level of MCM5 (*R*^2^ = 0.0004, *P* = 0.8) and CDC45 (*R*^2^ = 0.0001, *P* = 0.89) has no obvious correlation with the mRNA level of MINA53 (supplemental data 3). These data are to some extent consistent with our in vitro data. In vivo environment is much more complex than in vitro, the expression of CDC45, MCM2, MCM3, MCM5, ATM, and ATR may not only be affected by MINA53, but also altered by other regulating factors.

**Fig. 6:**
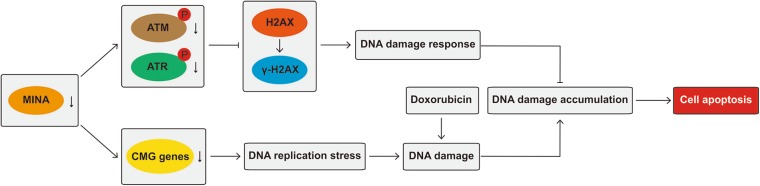
**Schematic illustration of the mechanism by which MINA53 deficiency induces DNA replication stress, diminishes DNA damage response and thus leads glioblastoma cell to apoptosis and death**

In summary, our study demonstrates that MINA53 is involved in DNA replication regulation and DNA damage response. Knockdown of MINA53 in glioblastoma cells downregulates the expression of the CMG genes and thus induces DNA replication stress. MINA53 knockdown further inhibits DNA damage response by reducing the ATM/ATR-H2AX pathway activity and leads glioblastoma cells to apoptosis and death (Fig. 6). And meaningfully, MINA53 deficiency could make glioblastoma cells more sensitive to Doxorubicin. These findings reveal the role of MINA53 in DNA replication and DNA damage response and suggest MINA53 as a novel and promising molecular target for glioblastoma therapy.

## Electronic supplementary material

Supplymentary figure legends

Supplementary Tables

Supplementary Fig.S1

Supplementary Fig.S2

Supplementary Fig.S3
