# Supplementary material for: MINA53 deficiency leads to glioblastoma cell apoptosis via inducing DNA replication stress and diminishing DNA damage response
Source: Cell Death Dis. 2018 Oct 17;9(11):1062. doi: 10.1038/s41419-018-1084-x (PMC6193027; doi:10.1038/s41419-018-1084-x)
Supplement: Supplementary file 1 — Supplymentary figure legends [file 41419_2018_1084_MOESM1_ESM.doc]

**Supplementary Figure legends**

**Fig. S1.** Western blot analysis of the indicated proteins in LN-229 and U-87 MG cells with or without MINA53 knockdown.

**Fig. S2.** Plot analysis to compare the indicated genes expression using two TCGA databases: GBM (n=154) and Brain Lower grade glioma (n=530).

**Fig. S3.** Linear regression analysis of the relevancy of MINA53 expression with CDC45, MCM2, MCM3, MCM5, ATM and ATR expression. Data was downloaded from TCGA GBM database (n=154) and analysed with GraphPad Prism 6.

**Tables**

**Table S1**. The sequences of primers used for qRT-PCR.

**Table S2**. The sequences of primers used for ChIP analysis.
