# Supplementary material for: MINA53 deficiency leads to glioblastoma cell apoptosis via inducing DNA replication stress and diminishing DNA damage response
Source: Cell Death Dis. 2018 Oct 17;9(11):1062. doi: 10.1038/s41419-018-1084-x (PMC6193027; doi:10.1038/s41419-018-1084-x)
Supplement: Supplementary file 2 — Supplementary Tables [file 41419_2018_1084_MOESM2_ESM.docx]

**Supplementary Table S1.** Primers used for qRT-PCR

| **Gene Name** | **Sequence (5'-3')** |
| --- | --- |
| MINA-f | CCAAAGAACTGCTTTCCTCAGAC |
| MINA-r | CTACACTGTCCAGCCTCGGTAA |
| CDC45 -f | TGGATGCTGTCCAAGGACCTGA |
| CDC45 -r | CAGGACACCAACATCAGTCACG |
| MCM2 -f | TGCCAGCATTGCTCCTTCCATC |
| MCM2 -r | AAACTGCGACTTCGCTGTGCCA |
| MCM3 -f | CGAGACCTAGAAAATGGCAGCC |
| MCM3 -r | GCAGTGCAAAGCACATACCGCA |
| MCM4 -f | CTTGCTTCAGCCTTGGCTCCAA |
| MCM4 -r | GTCGCCACACAGCAAGATGTTG |
| MCM5 -f | GACTTACTCGCCGAGGAGACAT |
| MCM5 -r | TGCTGCCTTTCCCAGACGTGTA |
| MCM6 -f | GACAACAGGAGAAGGGACCTCT |
| MCM6 -r | GGACGCTTTACCACTGGTGTAG |
| MCM7 -f | GCCAAGTCTCAGCTCCTGTCAT |
| MCM7 -r | CCTCTAAGGTCAGTTCTCCACTC |
| GINS1 -f | GCAAAGTCAGGTGGACGAAGTG |
| GINS1 -r | CTGATCCGAAGCAAGCGGTCAT |
| GINS2 -f | AGCCAAACTCCGAGTGTCTGCT |
| GINS2 -r | CTTGTGTGAGGAAAGTCCCGCT |
| GINS3 -f | GCTCCTGCATTTTGACAGTCCC |
| GINS3 -r | TCCATCTCGTCTAGCCTGGCTA |
| GINS4 -f | CTGGAGAGCAAGCCTGAGATTG |
| GINS4 -r | GCAAGTAGCTGCTGAGGACGTA |

**Supplementary Table S2.** Primers used for ChIP analysis

| **Gene Name** | **Sequence (5'-3')** |
| --- | --- |
| CDC45 -980~-849 -f | TGCTTCCAAAGGGATTGTAGG |
| CDC45 -980~-849 -r | CGGTGTTGTGTTCGGTAATGA |
| MCM2 -1616~-1515 -f | CACGCCCTTGAGTCCCATAC |
| MCM2 -1616~-1515 -r | AAGGGGGAAGAAGATGAGTTTGT |
| MCM3 -672~-575 -f | GGGGGCAGGGTAAACACAG |
| MCM3 -672~-575 -r | CCATTTGCTATCTGATACCGTTG |
| MCM5 -1341~-1189 | CTAGAGAAGCGGGATCTTGCTAT |
| MCM5 -1341~-1189 | GGGCTCCCTTCACTTCTAAGC |
