# Supplementary figures and images for: MINA53 deficiency leads to glioblastoma cell apoptosis via inducing DNA replication stress and diminishing DNA damage response
Source: Cell Death Dis. 2018 Oct 17;9(11):1062. doi: 10.1038/s41419-018-1084-x (PMC6193027; doi:10.1038/s41419-018-1084-x)

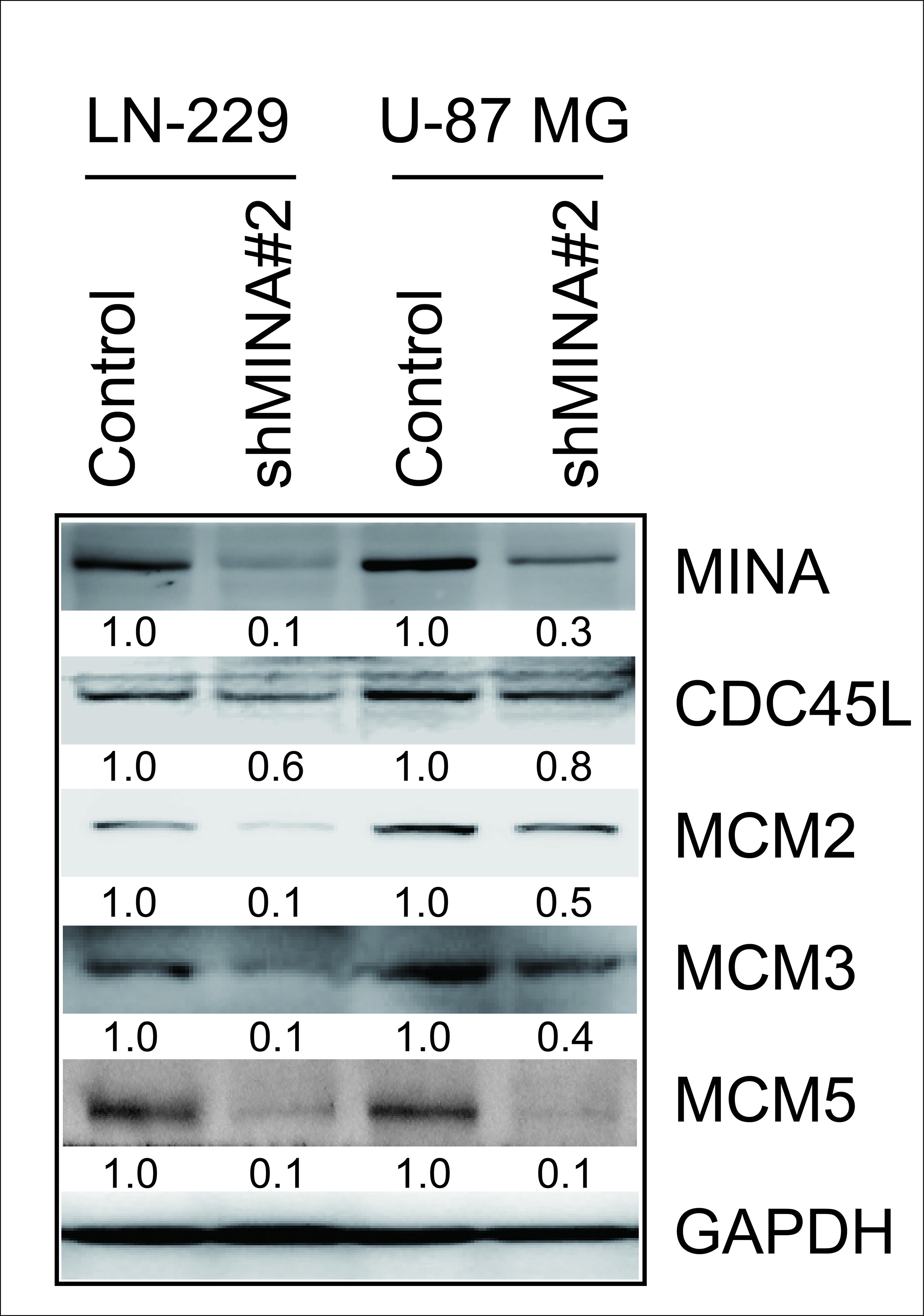

Supplement: Supplementary file 3 — Supplementary Fig.S1 [file 41419_2018_1084_MOESM3_ESM.jpg]

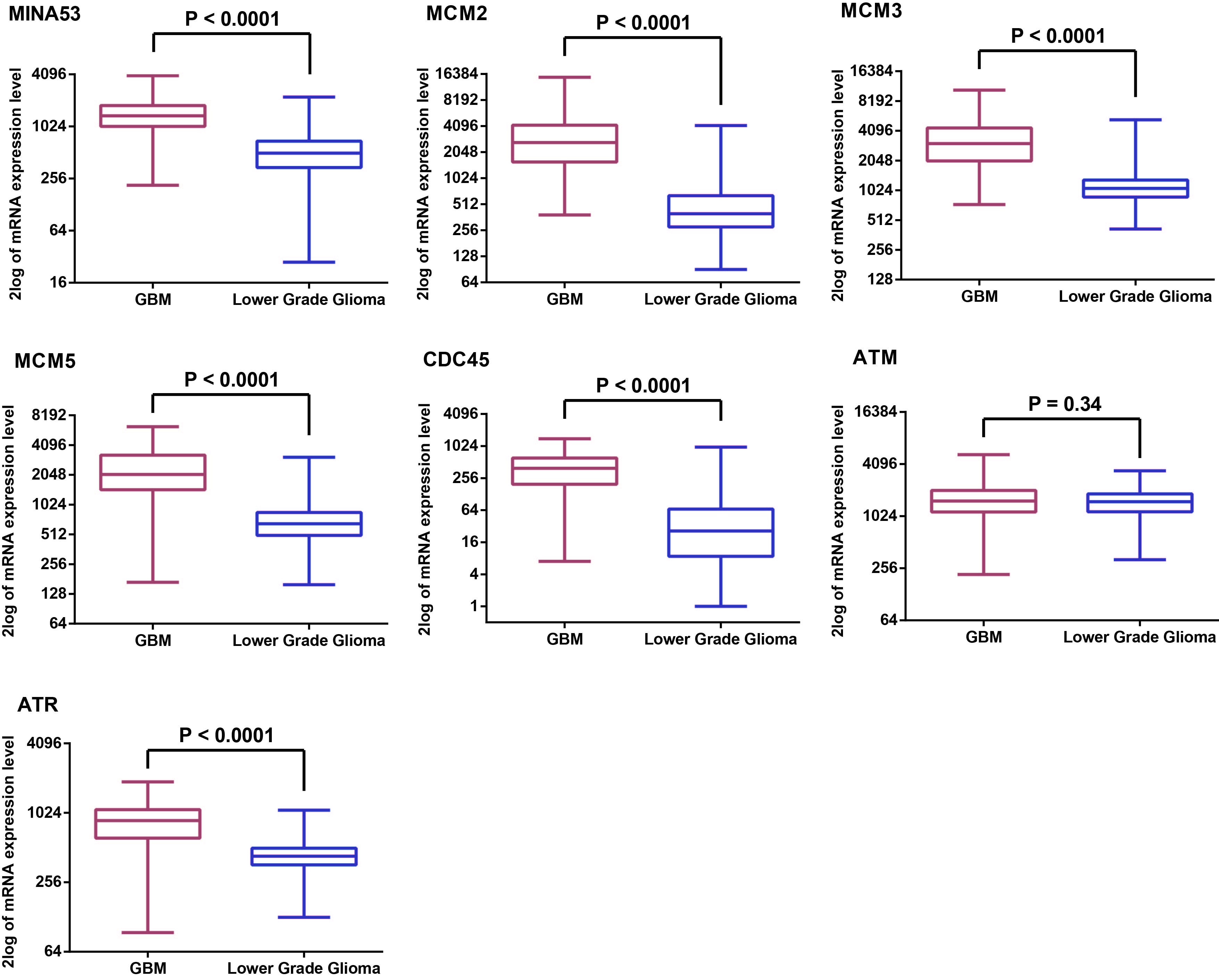

Supplement: Supplementary file 4 — Supplementary Fig.S2 [file 41419_2018_1084_MOESM4_ESM.jpg]

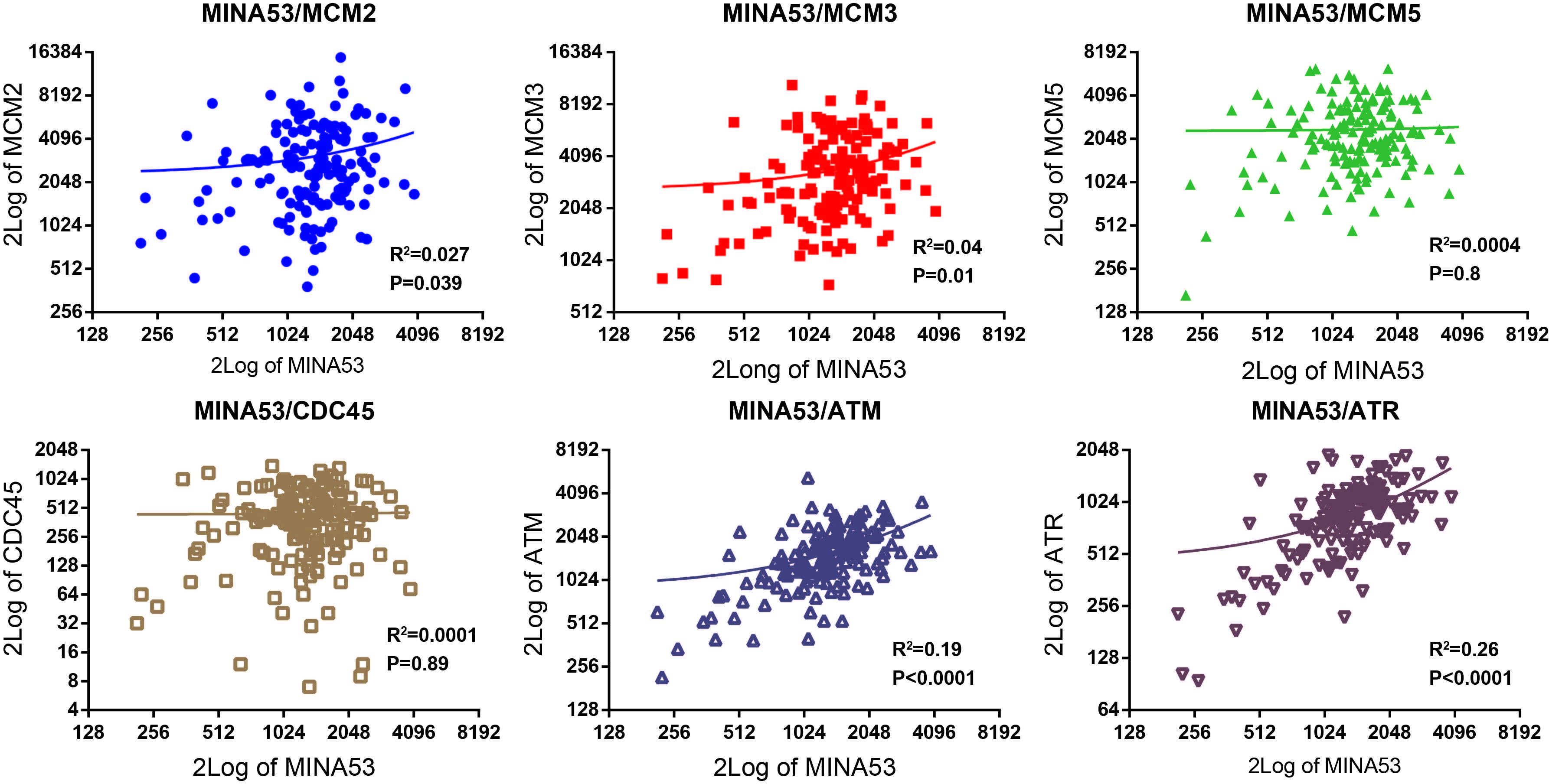

Supplement: Supplementary file 5 — Supplementary Fig.S3 [file 41419_2018_1084_MOESM5_ESM.jpg]
